# Aβ Oligomer Dissociation Is Catalyzed by Fibril Surfaces

**DOI:** 10.1021/acschemneuro.4c00127

**Published:** 2024-05-24

**Authors:** Alexander J. Dear, Dev Thacker, Stefan Wennmalm, Lei Ortigosa-Pascual, Ewa A. Andrzejewska, Georg Meisl, Sara Linse, Tuomas P. J. Knowles

**Affiliations:** † Biochemistry and Structural Biology, Lund University, Lund 221 00, Sweden; ‡ Centre for Misfolding Diseases Department of Chemistry, University of Cambridge, Lensfield Road, Cambridge CB2 1EW, U.K.; § Department of Applied Physics, Biophysics Group, SciLifeLab, Royal Institute of Technology-KTH, Solna 171 65, Sweden; ∥ Cavendish Laboratory, University of Cambridge, J J Thomson Avenue, Cambridge CB3 0HE, U.K.

**Keywords:** Alzheimer’s, oligomer, dissociation, kinetics, fibrils, therapeutic, inhibitor

## Abstract

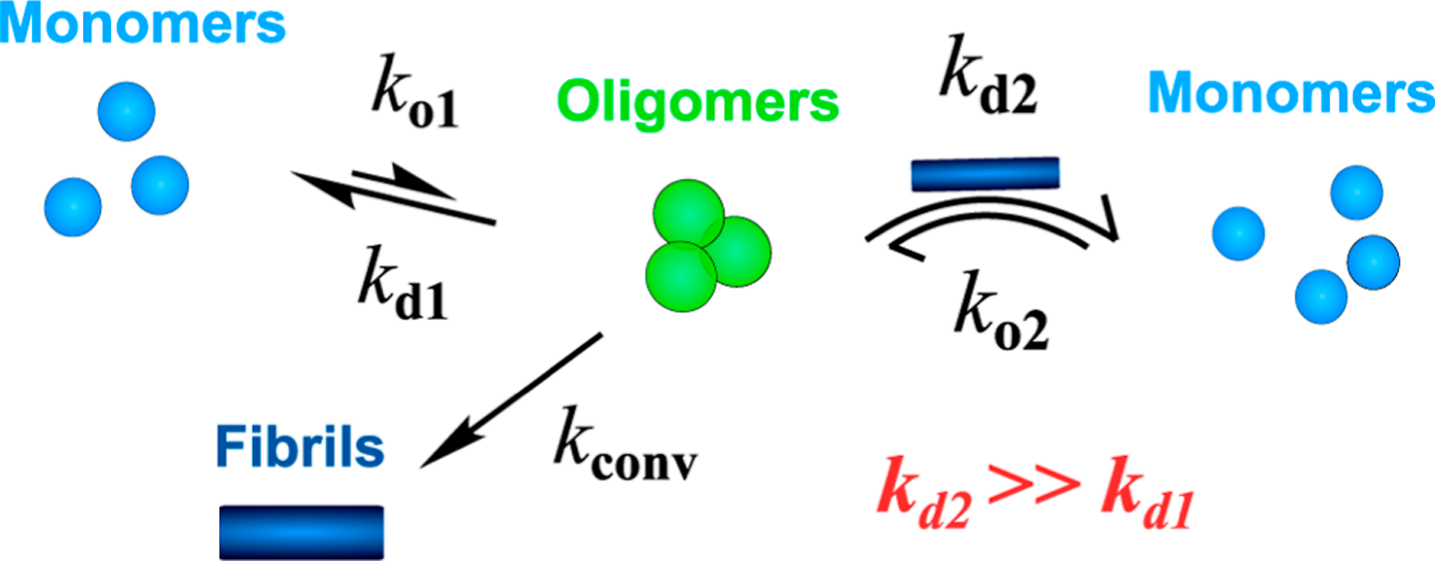

Oligomeric assemblies consisting of only a few protein subunits are key species in the cytotoxicity of neurodegenerative disorders, such as Alzheimer’s and Parkinson’s diseases. Their lifetime in solution and abundance, governed by the balance of their sources and sinks, are thus important determinants of disease. While significant advances have been made in elucidating the processes that govern oligomer production, the mechanisms behind their dissociation are still poorly understood. Here, we use chemical kinetic modeling to determine the fate of oligomers formed in vitro and discuss the implications for their abundance in vivo. We discover that oligomeric species formed predominantly on fibril surfaces, a broad class which includes the bulk of oligomers formed by the key Alzheimer’s disease-associated Aβ peptides, also dissociate overwhelmingly on fibril surfaces, not in solution as had previously been assumed. We monitor this “secondary nucleation in reverse” by measuring the dissociation of Aβ42 oligomers in the presence and absence of fibrils via two distinct experimental methods. Our findings imply that drugs that bind fibril surfaces to inhibit oligomer formation may also inhibit their dissociation, with important implications for rational design of therapeutic strategies for Alzheimer’s and other amyloid diseases.

## 1 Introduction

Amyloid oligomers are small clusters of protein molecules noncovalently bound together and are intermediates of plaque and prion self-assembly.^1,2^ They cause cell death and are the chief pathogenic agents in many of the most severe health challenges faced by society today, such as Alzheimer’s and Parkinson’s diseases.^3−12^ In particular, a key upstream process in Alzheimer’s disease (AD) is the self-assembly of Aβ40 and Aβ42 peptides into amyloid oligomers.^13^ However, until very recently, little was known about the mechanisms by which amyloid oligomers are created and destroyed. This lack of knowledge has hindered our understanding of the molecular origins of these diseases and is fatal to rational drug design efforts, as indicated by the failure of hundreds of Alzheimer’s drugs in clinical trials over the past 2 decades.^14−16^

**Figure 1:**
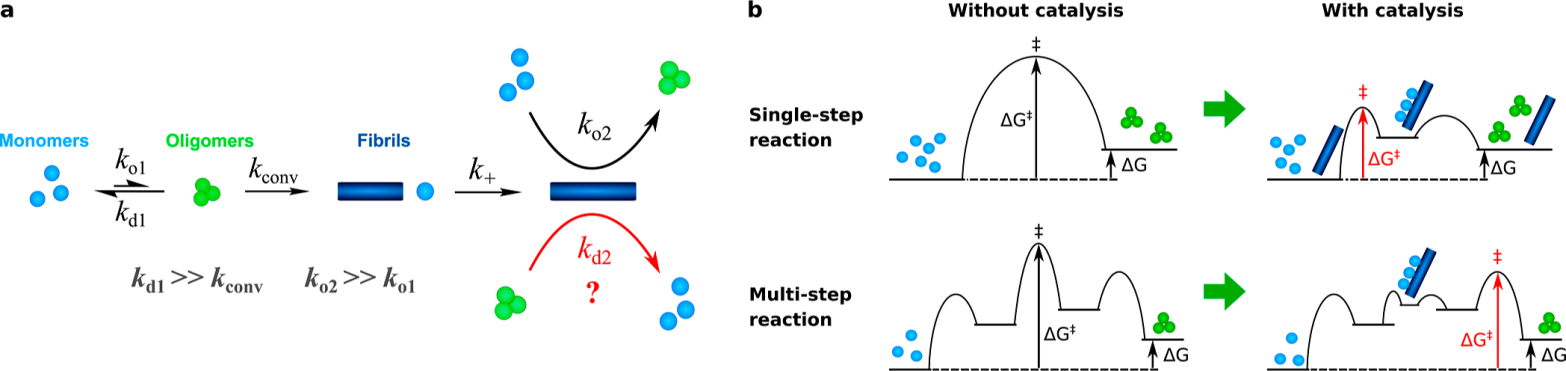
(a) Coarse-grained reaction steps in amyloid oligomer and fibril assembly kinetics (rate constants in brackets). Nonfibrillar oligomers are formed by free association of monomers (“primary association”, *k*_o1_) and subsequently undergo conformational conversion into fibrils (*k*_conv_) that rapidly elongate via addition of monomers to their ends (*k*_+_). However, the majority of oligomers dissociate back to monomers (*k*_d1_), rather than convert to fibrils. In Aβ aggregation, nonfibrillar oligomers are also formed through association on the surfaces of fibrils (*k*_o2_). For all but the lowest fibril concentrations, this occurs much more rapidly than the primary association of oligomers. However, the extent to which oligomers also dissociate at the surface of fibrils (*k*_d2_) has not hitherto been determined. (b) Catalytic effect of fibrils on hypothetical single step and multistep oligomerization reactions. In the absence of fibrils, Aβ oligomer formation and dissociation are both slow. Oligomers are less thermodynamically stable than monomers. In the presence of fibrils, the relative thermodynamic stabilities of monomers and oligomers (Δ*G*) are unchanged, but oligomerization is greatly accelerated. In a single-step reaction, the only energy barrier present is reduced in height by the fibril surface catalyst. Therefore, both oligomer formation and dissociation are accelerated equally. In a multistep reaction, the forward and backward rates for the step that is catalyzed increased equally. However, if enough catalysis occurs, then the greatest energy barrier is now different. The free energy barrier Δ*E*^‡^ dividing the species is reduced equally, but the Arrhenius-type prefactor will be affected differently in the forward and reverse directions. Thus, formation and dissociation of oligomers are still both accelerated by fibrils but to slightly different extents.

The development in the mid-2010s of techniques to monitor oligomer concentrations accurately throughout aggregation reactions^17^ has enabled quantitative modeling of oligomer kinetics and driven a rapid advancement in our understanding of the coarse-grained reaction steps involved (Figure 1a). The direct association of monomeric proteins to form new oligomers is referred to here as “primary association”. They may subsequently undergo slow conformational or structural conversion to form rapidly growing fibril nuclei or possibly other nonfibrillar oligomeric species.^18,19^ The overall formation of fibril nuclei from monomers via this pathway is widely referred to as “primary nucleation”. It was originally assumed that oligomer formation is effectively irreversible on the time scale of typical in vitro aggregation experiments because in the earliest studies, only α-synuclein oligomers were studied,^19^ which dissociate back to monomeric protein extremely slowly relative to the experimental time scale employed. However, later studies of other systems^20−22^ found oligomer dissociation to be a crucial step in the kinetic model and indeed to be much faster than their conversion to fibril nuclei. Subsequently, a meta-analysis of earlier oligomer kinetic studies revealed that all oligomer populations hitherto monitored over time dissociate faster than they convert to fibrils,^23^ including α-synuclein oligomers, suggesting that this is a universal property of the most populous oligomeric species. Around the same time, the kinetics of the total Aβ42 (and Aβ40) oligomer populations were measured. Their successful modeling required a single additional reaction step: formation of oligomers by the interaction of monomers with the surfaces of fibrils (“secondary association”) and occurring much more rapidly than primary association.^24^ Evidence indicates that they are on-pathway to fibril formation;^2,24^ the overall production of new fibril nuclei from monomers via the conformational conversion of these oligomers is known as “secondary nucleation”.

Again, a majority of both Aβ40 and Aβ42 oligomers were found to dissociate much faster than they converted to fibrils. However, to date, it has not been explored precisely how this dissociation occurs. In particular, since Aβ oligomer formation has been shown to most frequently occur by catalysis at fibril surfaces, does this mean the same is true for oligomer dissociation (Figure 1a)? This question is of critical importance because since dissociation is the chief reaction responsible for destruction of oligomers in vitro, the mechanism and rate of dissociation determine not only the lifetime of these toxic species but also their abundance.^23^ An answer is therefore likely needed to understand the pathology of AD and other amyloid diseases in which secondary association is shown to play a role. Here, we use a combination of theory and experiment to answer this question in the affirmative. We demonstrate that fibril-mediated oligomer dissociation has fundamental implications for the kinetics of Aβ aggregation under the near-steady-state conditions likely to be found in vivo. We subsequently explore the potentially profound consequences for the rational design of therapeutics for AD.

## 2 Results

### 2.1 Fibrils Strongly Catalyze Aβ42 and Aβ40 Oligomer Formation

Our first claim that fibril surfaces are catalysts for Aβ42 and Aβ40 oligomer formation is not a controversial one as it is a consequence of very basic principles of physical chemistry. Indeed, fibril surfaces have been repeatedly referred to as catalysts for new Aβ40/42 *fibril* formation without attracting controversy.^25−27^ The long-established definition of a catalyst can be found in the authoritative IUPAC Compendium of Chemical Terminology.^1^ In abbreviated form, it reads: “a substance that increases the rate of a reaction without modifying the overall standard Gibbs energy change in the reaction and is both a reactant and product”. Importantly, this definition is completely agnostic as to whether the reaction is a single step or multistep one.

Now, fibril surfaces are clearly both reactants and products of oligomer formation via secondary association. Moreover, the Gibbs energy change Δ*G* is given by the relative stabilities of the reactants and products; here, these are free monomers and free oligomers. Since these reactant and product species are unchanged by the availability of fibril surfaces (assuming that the same oligomeric species are produced via primary and secondary association), Δ*G* is unchanged.

Alternatively, if the oligomers formed via secondary association are different chemical species from those formed through primary association, our argument is only strengthened. In this case, the “secondary oligomers” would be the overwhelming majority species, and the catalytic effect of fibrils on their formation would be even greater. This follows since their formation rate in the absence of fibrils must by definition be far lower than the rate of formation of “primary oligomers”. So, the second and third criteria of the definition are satisfied.

Next, the extent to which fibrils accelerate oligomer formation, *a*, can be quantified as the ratio of the rates for oligomer formation through secondary and primary association. Determination of these rates requires model fitting to kinetic data on oligomer concentrations, which are much less widely available and more difficult to collect than data for the fibril mass concentration. It is therefore more convenient to write *a* in terms of the rate constants for *fibril* formation through secondary and primary nucleation, *k*_2_ and *k*_*n*_ respectively, as (Materials and Methods)
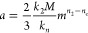
1where *m* is the concentration of monomeric protein, *M* is the mass concentration of fibrils, and *n*_c_ and *n*_2_ are the reaction orders with respect to monomer concentration for fibril formation through primary and secondary nucleation. (Clearly, if the oligomers produced via primary and secondary association were different species, *a* would instead be a lower bound on the catalytic effect.) These parameters have been determined accurately for many different reaction conditions and peptides in earlier studies.

Now, it has been established across multiple studies of both Aβ42 and Aβ40 that *n*_c_ ≃ *n*_2_ ≃ 2. Using the kinetic parameters found in refs (25 and 26), we may thus calculate the acceleration of oligomer formation as *a* = *xM*, where *x*(Aβ42) ≃ 30 μM^–1^ and *x*(Aβ40) ≃ 1100 μM^–1^. As just one example, if *M* = 1 μM, the accelerations will amount to *a*(Aβ42) = 30 and *a*(Aβ40) = 1100. Thus, the rate of the reaction is clearly greatly increased, and the first criterion for a catalyst is fulfilled for both Aβ42 and Aβ40. Intriguingly, although the overall kinetics are not much different from wild-type Aβ42, the ratio of *k*_2_/*k*_*n*_ is increased ca. 100-fold for the peptide with the familial mutation A2 V, making the catalysis of oligomer formation 100 times more efficient than that for the wild type.^28^

### 2.2 Strong Catalysis of Oligomer Formation Implies Strong Catalysis of Oligomer Dissociation

Our next claim is that under conditions where they strongly catalyze oligomer formation, fibril surfaces must also strongly catalyze oligomer dissociation. If oligomer formation were best modeled as a single-step reaction, this would obviously be true: a catalyst must increase equally both forward and reverse rates. This is because catalysts modify the barrier height, and since this enters into the exponent of the Arrhenius equation for the rate constants, a given reduction in barrier height leads to the same enhancements of both the forward and backward rate constants. However, catalysts leave the start and end states, and thus the equilibrium constant *K*, unchanged (Δ*G*° = −*RT* ln *K*).^29^ In a single-step reaction *K* = *k*_f_/*k*_b_, where *k*_f_ and *k*_b_ are the forward and backward rate constants. Thus, any increase in the forward rate must be exactly matched by an increase in the reverse rate (Figure 1b).

Formation of protein oligomers from monomers features many conformational and solvational changes, as well as direct interactions between monomers. It is thus possible that metastable intermediates exist, as do multiple energy barriers, in which case it is best described as a multistep reaction. A catalyst then typically only reduces the height of one of the energy barriers (Figure 1b). Overall, oligomer formation and dissociation are described by coarse-grained rate laws that in general, change upon large changes in the concentrations of the various species in the multistep reaction. Therefore, the equilibrium constant is not, in general, the ratio of the coarse-grained rate constants, admitting the possibility that the coarse-grained forward and backward rates do not increase equally in the presence of fibrils.

However, even if oligomerization is best described as a multistep reaction, the strongest determinant by far of these coarse-grained rate constants is still the height of the largest free energy barrier.^30^ If fibril surfaces cause a strong increase in the oligomerization rate, they must significantly reduce the height of the largest barrier and thereby also cause a strong increase in dissociation under the same reaction conditions. This increase may not be exactly identical because the catalyst can change the identity of the highest energy barrier in a multistep reaction (Figure 1b), so changes in the free energy landscape may not be the same either side of this barrier. However, these changes are of only secondary importance to the barrier height in determining the overall rate. Therefore, we can conclude that the increase in the oligomer dissociation rate is approximately as large as the increase in the formation rate at the same monomer concentration. Using eq 1, we can finally convert this conclusion into algebra, and write
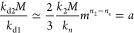
2where *k*_d1_ and *k*_d2_ are the rate constants for free and fibril-mediated dissociation, respectively.

### 2.3 Almost All Oligomeric Intermediates of Aβ42 and Aβ40 Fibril Self-Assembly Dissociate at Fibril Surfaces

**Figure 2:**
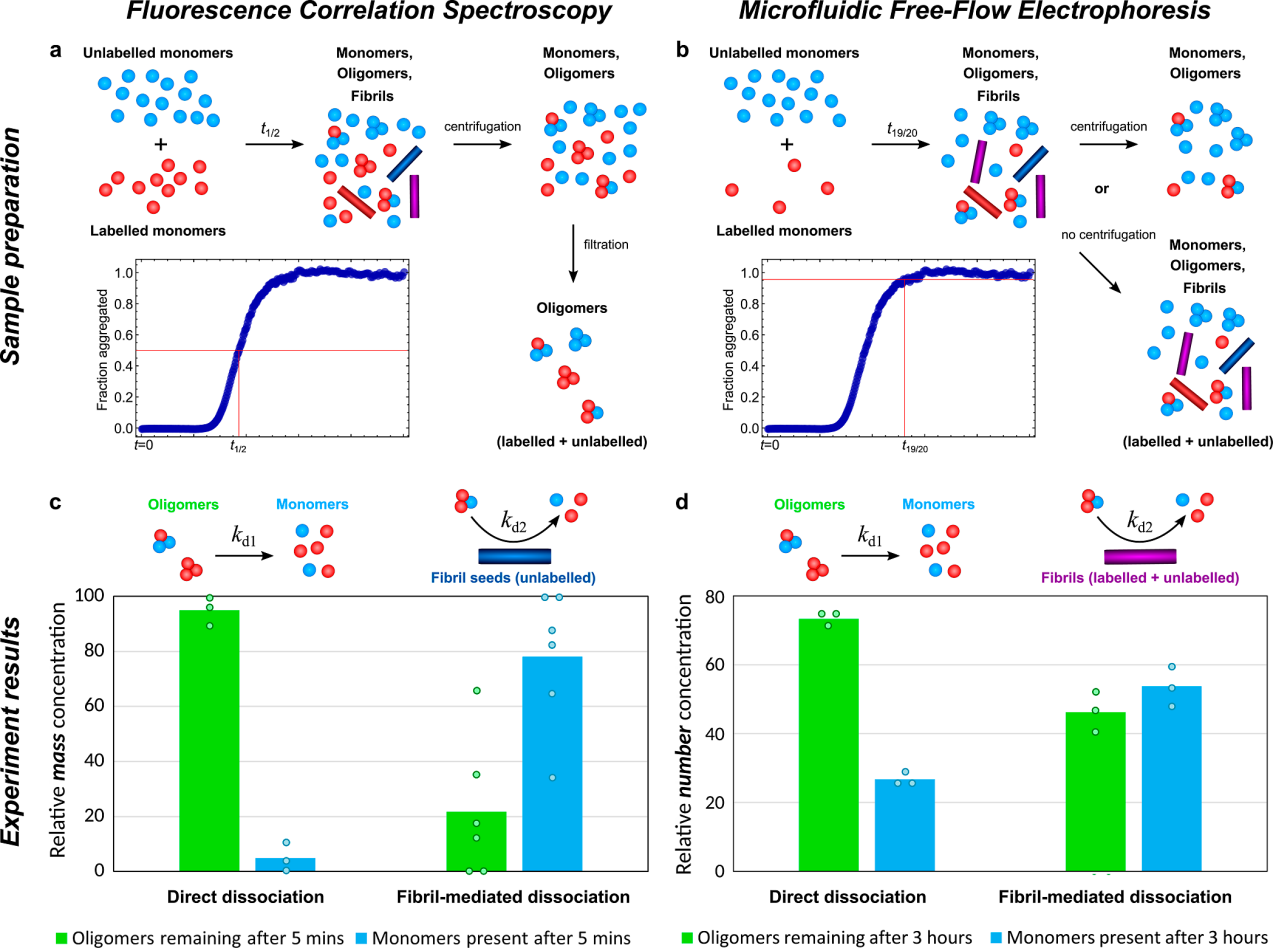
Two independent experiments with different methodologies confirm that fibrils catalyze oligomer dissociation. Fluorescently labeled and unlabeled monomeric protein with a molar ratio of 1:1.5 (a) or 1:3.5 (b) was incubated until c. 50% (a) or c. 95% (b) of monomers have formed fibrils. (a) For fluorescence correlation spectroscopy (FCS), all samples were then centrifuged to remove most fibrils, and unlabeled fibril seeds were added back in to some of the samples. (b) For microfluidic free-flow electrophoresis (μFFE), only some samples were centrifuged, with the uncentrifuged samples thus effectively containing labeled seeds. (c) FCS measurements of the *mass* concentration of oligomers and monomers were taken after approximately 5 min of incubating oligomers ± fibrils. (d) μFFE measurements of the *number* concentration of oligomers and monomers were taken after approximately 3 h of incubating oligomers ± fibrils. In both cases, relative labeled oligomer concentrations are much lower when fibrils are present (due to both a decrease in absolute oligomer concentrations and an increase in absolute monomer concentrations), showing that fibrils cause many more oligomers to dissociate in the first 5 min and that this effect holds under very different labeling conditions. The bars represent means over the experiment repeats. *t*-tests confirmed the significance of this decrease (*p* < 0.001 in both experiments, see Materials and Methods).

In a typical aggregation experiment, most Aβ42 and Aβ40 oligomer dissociation occurs at the end of the reaction, when there are few monomers and many fibrils. Our final claim, that almost all oligomer dissociation occurs at fibril surfaces, can thus be tested using eq 2 with appropriate monomer and fibril concentrations. However, we first validated our claim experimentally for Aβ42. Alexa-labeled Aβ42 oligomers were produced under two distinct sets of conditions, both related to those of prior kinetic studies (Figure 2a,b; Materials and Methods).

The oligomers formed under the conditions illustrated in Figure 2a were isolated by centrifugation and filtration and added to a fresh buffer solution both with and without unlabeled fibrils. After approximately 5 min of incubation, their mass concentration was inferred from FCS (see Materials and Methods), as was that of monomers. When incubated in the presence of fibrils, the oligomer mass concentration was found to be significantly lower than that in their absence (Figure 2c), indicating that oligomer dissociation is greatly accelerated by fibrils. The strong catalytic role of fibrils was confirmed by the concentration of labeled monomers, that can only be produced by oligomer dissociation, being several times higher in the presence of fibrils. Note that although Alexa-labeled Aβ42 displays qualitatively similar kinetics to unlabeled Aβ42, the overall time scale of Alexa-labeled fibril formation is longer due to monomer stabilization by the label.

Only half of the oligomer samples produced under the conditions illustrated in Figure 2b were centrifuged to remove fibrils, and all samples were then incubated for 3 h. The number concentrations of monomers and oligomers were then measured by μFFE (see Materials and Methods). In the uncentrifuged samples that contain labeled fibrils, the relative oligomer concentration was found to be significantly lower (Figure 2d), indicating their dissociation to be greatly accelerated by fibrils. This was again due to both a decrease in oligomer concentration and an increase in monomer concentration, confirming the strong catalytic role of fibrils. Note that the numbers are not comparable between these two experiments due to a number of differences in methodology; however, these differences support the robustness of our findings. The measurement times were chosen to be as soon as possible after sample collection while still providing sufficient time for sample processing and data collection and for the difference in oligomer dissociation rates with and without fibrils to become detectable.

These experimental results are in line with our above discussion that there is likely only one large barrier in the free energy landscape, and moderate changes in concentrations are not likely to change the greatest energy barrier to one unsusceptible to catalysis by fibril surfaces. This is particularly true in the case of Aβ42^25^ and Aβ40,^26^ for both of which it has been established that *n*_c_ ≃ *n*_2_ ≃ 2 over a wide range of monomer concentrations, meaning that the catalytic effect of fibrils on both Aβ42 and Aβ40 oligomer dissociation (and formation) is largely *independent* of monomer concentration. For these peptides, eq 2 gives *k*_d2_*M*/*k*_d1_ = 30·*M* for Aβ42 and 1100·*M* for Aβ40, verifying that this catalytic effect is substantial for both peptides.

**Figure 3:**
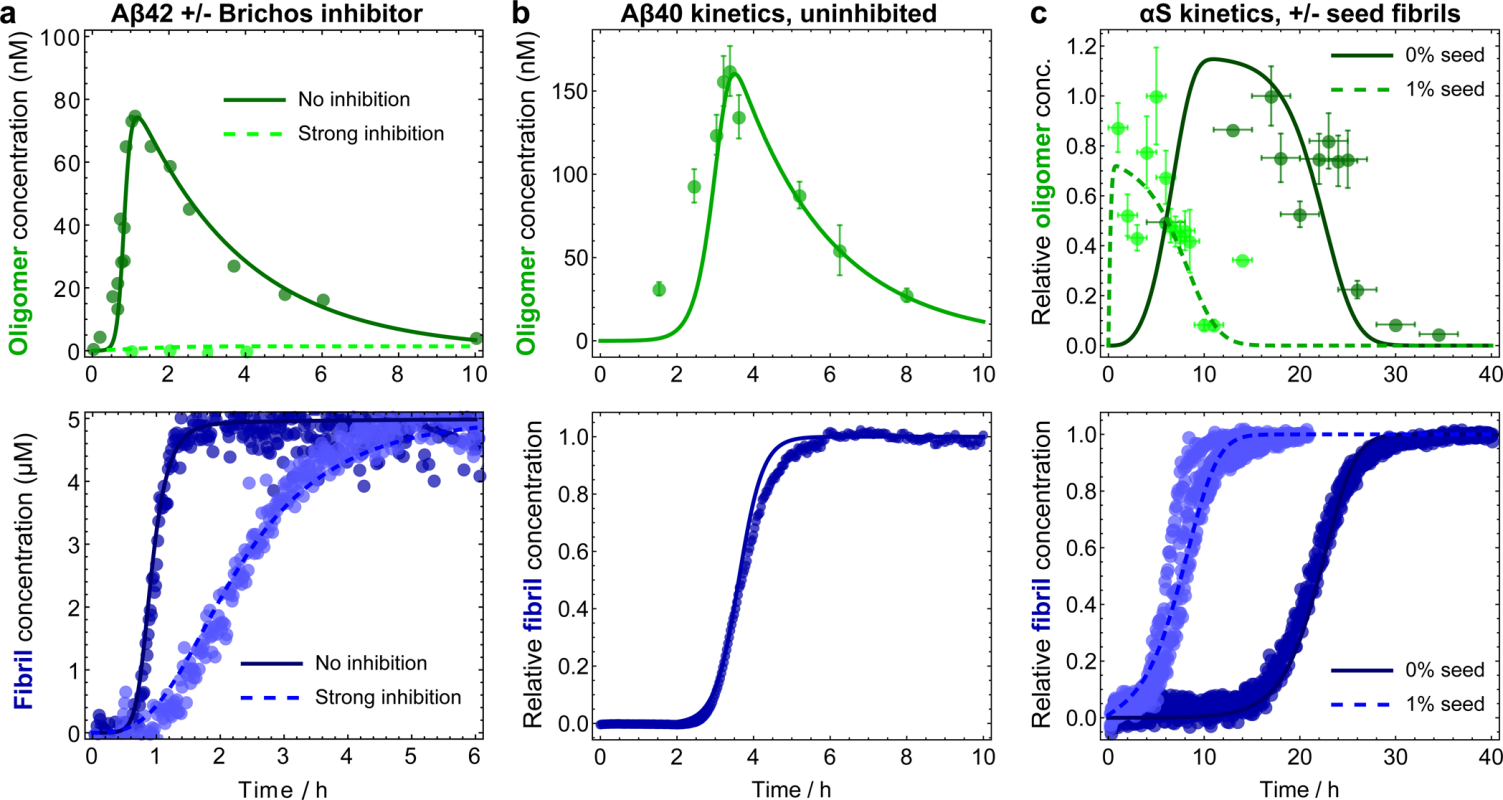
Fibril-mediated oligomer dissociation is consistent with the available kinetic data on amyloid proteins that undergo rapid secondary nucleation. Top: oligomer concentration and bottom: fibril concentration. Global fits are to kinetic models in which the great majority of oligomers dissociate on fibrils. (a) Aβ42 oligomer-mediated fibril formation kinetics (total monomer concentration *m*_tot_ = 5 μM) both in the presence and absence of a known inhibitor of oligomer formation through secondary nucleation (data taken from ref (24); most rate constants unchanged). (b) Aβ40 oligomer-mediated fibril formation kinetics (*m*_tot_ = 10 μM; data again taken from ref (24); most rate constants unchanged). (c) α-Synuclein oligomer-mediated fibril formation kinetics in both the presence and absence of 1 μM fibril seed (*m*_tot_ = 100 μM; data and rate constants taken from ref (31)).

Consistency with previous kinetic experiments of this new mechanism for oligomer dissociation was verified by refitting the data from ref (24) on Aβ40 and Aβ42 oligomer formation during fibril self-assembly (Figure 3a,b). The only adjustment to the rate constants needed is found to be a reduction in the dissociation rate constant by a factor equal to the final fibril mass concentration. The resultant fits are no worse than those obtained via the original model involving free oligomer dissociation, as shown in ref (24). This follows because in these systems, oligomer dissociation is slow compared to fibril formation, so differences in the dissociation rate before the half time of fibril formation are relatively inconsequential.

Figure 3c also reproduces the data and fits from ref (31); that paper already assumed fibril-mediated oligomer dissociation, so no refitting was needed. It demonstrates that the main effect of adding preformed fibril seeds on oligomers in systems with secondary nucleation is to cause them to appear earlier in the reaction due to an increase in initial oligomer formation rate. Since dissociation is increased proportionally, the equilibrium oligomer concentration is unaffected. (Note that absolute oligomer concentrations were not measured by the experimental technique used in Figure 3c; the apparent reduction in oligomer concentration upon adding seed is an artifact of the data normalization.)

### 2.4 Under Constant-Monomer Conditions, Aggregation Kinetics Are Radically Altered by Fibril-Mediated Dissociation

In living organisms, proteostasis keeps a variety of proteins at a relatively constant monomer concentration. Thus, modeling the kinetics of protein aggregation when the monomer concentration is held constant is an important step toward understanding protein aggregation in vivo. Since under constant-monomer conditions, Aβ42 fibril formation does not end before oligomer dissociation gets underway, these processes are coupled.

**Figure 4:**
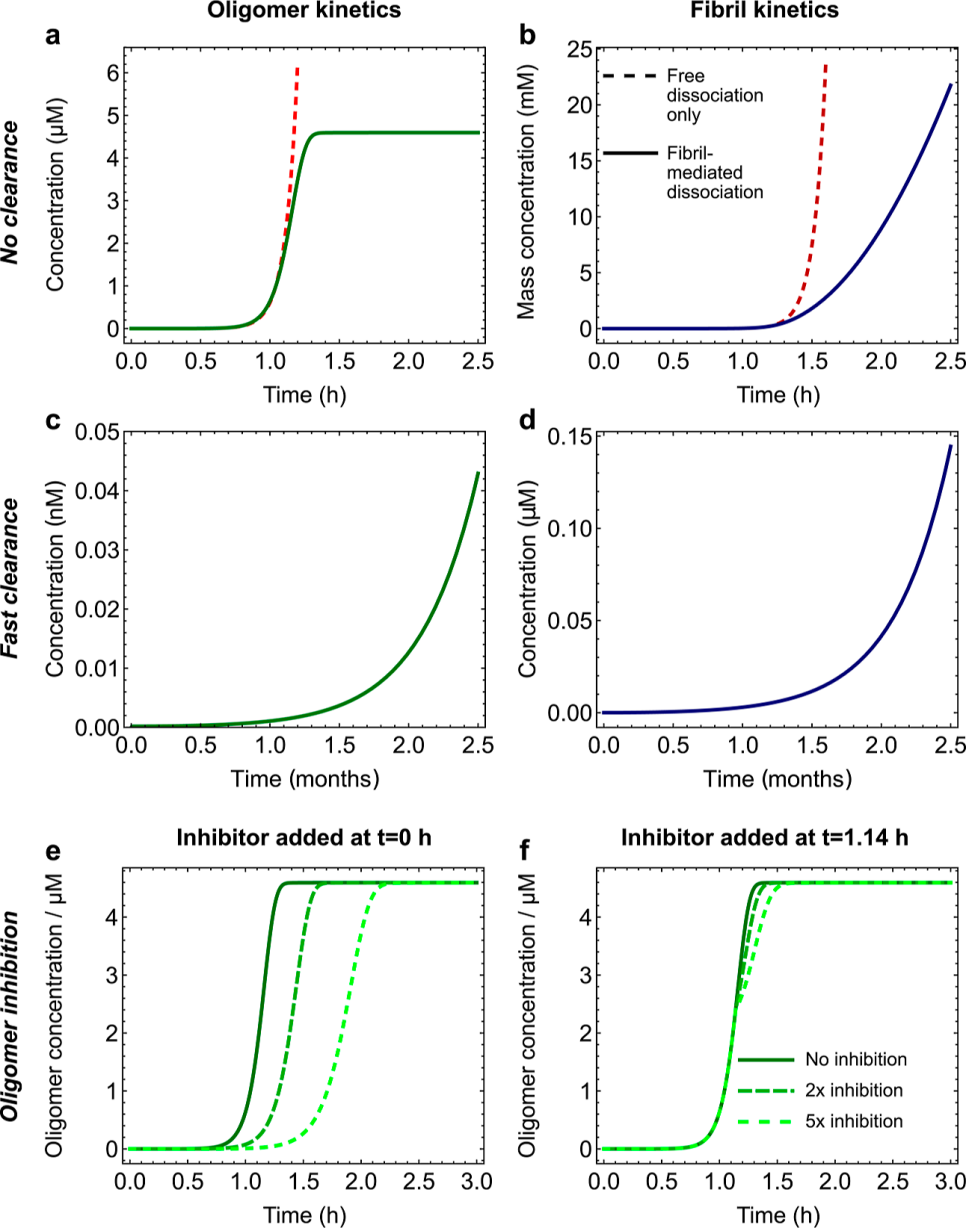
Effect of oligomer-mediated dissociation on Aβ42 aggregation kinetics under constant-monomer conditions, simulated using rate parameters from ref (24). (a,b) Constant *m* = 5 μM and no removal of oligomers. In the absence of fibril-mediated dissociation, both would rise exponentially indefinitely (red, dashed). However, we have found that dissociation is actually fibril-mediated. As a result, oligomer concentration (green) instead plateaus, whereas fibril mass concentration (blue) rises initially exponentially but subsequently as *t*^2^ once oligomer concentration plateaus. (c,d) Conditions closer to those expected in vivo with fast oligomer clearance mechanisms, i.e., *m* = 0.1 μM and *k*_cl_ = 25κ. No plateau is reached for several months. (e,f) Fibril-binding inhibitors must be added sufficiently early to reduce Aβ42 oligomer concentrations under constant-monomer conditions. Solid lines: no inhibition. Dashed lines: mild inhibition, *k*_I_*c*_d_ = 1. Dotted lines: strong inhibition, *k*_I_*c*_d_ = 4. (e) If the inhibitor is added at *t* = 0, it can greatly delay the time at which the plateau is reached but cannot affect the plateau concentration. (f) If the inhibitor is added at *t* = 1.14 h (approximately, the half-time for oligomer formation), it has little effect on the oligomer kinetics. If added after the plateau, inhibitors would have no effect at all, even at very high concentrations.

The kinetics of oligomer and fibril self-assembly with secondary association of oligomers but *without* fibril-mediated dissociation under constant-monomer conditions have been solved previously.^24,32^ It was found that both oligomer and fibril concentrations grow exponentially indefinitely once secondary association dominates over primary association (red dashed lines in Figure 4), with the exponent κ describing the effective proliferation rate of oligomers or fibrils. This occurs when *M* ≫ *M*_12_, where *M*_12_ is
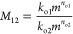
3

By contrast, when fibril-mediated dissociation is added to the kinetic model, the oligomer concentration *S* instead follows sigmoidal kinetics (Figure 4a and Materials and Methods), plateauing after an initial exponential phase at its steady-state value of *S*_ss_ = *k*_o2_*m*^*n*^_o2_/*k*_d2_, where *k*_o2_*m*^*n*^_o2_*M* is the rate of formation of oligomers via secondary association. Steady state is attained when the rate of oligomer proliferation is counterbalanced by oligomer dissociation. In living systems, the steady-state condition can take a more complex form as the removal of oligomers by cellular clearance processes has to be taken into account (see below). Under constant monomer conditions without oligomer removal, steady state occurs when κ*S* – *k*_d2_*MS* ≃ 0, i.e., oligomer production and removal rates cancel. So, for steady state to occur, the fibril mass concentration must exceed *M*_c_, given by
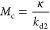
4

In Materials and Methods, we show that when *M* exceeds *M*_c_, the oligomer concentration is always within 37% of its steady-state value, confirming the suitability of this threshold value of *M* as an indicator of the achievement of steady-state conditions. At this point, the rate of nucleation of new fibrils becomes constant in time, so growth in fibril mass concentration transitions from exponential to quadratic (see Figure 4b and Materials and Methods).

### 2.5 Consequences for In Vivo Kinetics

The fact that preformed aggregates are capable of triggering aggregation in living systems,^33,34^ as well as the observation that the rate of secondary nucleation is predictive of involvement in pathology,^35^ suggests that during AD, the aggregate mass present in vivo exceeds *M*_12_ and that cytotoxic oligomer formation and dissociation both occur primarily on fibril surfaces. At low amyloid loads, as expected in the earliest stages of AD, oligomer dissociation nonetheless likely plays little role in determining oligomer populations because *M* < *M*_c_. In this regime, oligomer concentrations increase proportionally to the fibril concentrations. It is possible that amyloid loads eventually become high enough that *M* > *M*_c_ in late-stage AD. If so, fibril-mediated dissociation becomes the most important process governing oligomer loss, and the concentration of cytotoxic oligomers will no longer be correlated with the Aβ fibril concentration through direct biophysical mechanisms.

The value of *M*_c_ depends on monomer concentration *m*. In Figure 4a,b, we used a typical in vitro value of *m* = 5 μM. However, the average Aβ concentration in the human brain is likely nanomolar or even picomolar (although this value is likely to vary with location, and higher local concentrations are possible). In the hypothetical situation, where conditions are the same as those in standard in vitro experiments but the monomer concentration is kept constant throughout the reaction at *m* = 100 nM, we would obtain *M*_c_ ≃ 440 nM. However, as has been shown by studies of aggregation in cerebrospinal fluid^36^ and living systems,^37,38^ aggregation rates tend to differ significantly in vivo, although the fundamental mechanism of aggregation appears to generally be conserved. In addition, this estimation of *M*_c_ also neglects two crucial phenomena that occur in biological organisms and can greatly alter the kinetics of aggregate and oligomer formation: removal or clearance^39^ and transport.^38^ The former involves the active destruction of oligomers and aggregates; the latter is simply the removal of oligomers from areas of high oligomer production to areas of low amyloid plaque content. For an organism at steady state, the rate for these combined processes can be modeled as *k*_cl_*S*, encapsulating both removal of oligomers and transport away from their sites of production.

A necessary condition for the steady-state oligomer concentration to be attained is that fibril-mediated dissociation be the major mechanism for removal of oligomers, i.e., *k*_d2_*M* > *k*_d1_ + *k*_conv_*m*^*n*^_conv_ + *k*_cl_. In vitro this condition is generally satisfied when the fibril mass concentrations approach *M*_c_ = κ/*k*_d2_ because there is no clearance (*k*_cl_ = 0). In an in vivo context, when significant clearance may be present, we must modify the definition of *M*_c_ to
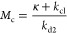
5

We can draw interesting conclusions from this. First, if clearance and transport play a prominent role in the removal of oligomers from their sites of production, then the threshold fibril mass concentration *M*_c_ above which they attain their steady-state concentration is elevated over the in vitro value. Second, *M*_c_ depends critically on the in vivo rates of bulk oligomer proliferation κ, oligomer clearance and transport from the location of their production *k*_cl_, and their fibril-mediated dissociation *k*_d2_. These have not been directly measured; however, evidence of a strong clearance mechanism and significantly reduced κ suggest that the steady-state fibril concentrations in vivo are likely to be significantly higher than their hypothetical in vitro counterparts. It is thus unclear if aggregate levels of *M*_c_ are ever exceeded in vivo and, thus, if oligomer concentrations reach a steady-state value under these conditions.

### 2.6 Consequences for Drug Design

An interesting consequence of fibril-mediated oligomer dissociation is that once the oligomer concentration has plateaued at its steady-state value, it is *unaffected* by drugs that bind fibril surfaces, such as Brichos. This is true regardless of the oligomer clearance or conversion rates and follows because the rates of both surface-catalyzed oligomer formation and surface-catalyzed dissociation are affected equally by the blocking of catalytic fibril surface by inhibitors. Drugs that inhibit oligomer formation by binding to fibrils will therefore only have a significant inhibitory effect on oligomers (and consequently on fibril accumulation) if the drug is administered while *M* < *M*_c_, and the oligomer concentration is still in the exponential growth phase (Figure 4e). At higher aggregate concentrations, the drug had little effect (Figure 4f). Note that *M*_c_ is also increased by the inhibitor due to its reduction of *k*_d2_. Naturally, fibril surface-binding drugs also have a significant inhibitory effect only when secondary association is a more important source of new oligomers than primary association. This requires *M* > *M*_12_. Therefore, the fibril concentration range in which these drugs are effective is given by *M*_12_ < *M* < *M*_c_.

The conclusion that fibril-binding drugs are only effective when the main source of oligomers is fibril-catalyzed association and when their main sink is *not* fibril-catalyzed dissociation should hold also in vivo and in disease. Exceeding *M*_c_ at high aggregate loads may be a contributing factor in the failure of Alzheimer’s drugs administered in the later stages of the disease. The task of determining when this is the case, i.e., estimating *M*_c_ and *M*_12_ in the brains of AD patients, is however nontrivial, as discussed above. Nonetheless, such estimations may not always be necessary. Rapid enough clearance may raise *M*_c_ above the values of *M* ever attained by the organism of interest, guaranteeing that binding fibril surfaces to inhibit oligomer formation remains an effective strategy even under high aggregate loads. Moreover, lowering *k*_d2_ (for instance, by applying such drugs) raises *M*_c_; sufficiently early treatment may therefore guarantee that the drug will remain effective indefinitely.

## 3 Discussion

In this study, we focused on dissociation of Aβ42 and Aβ40 oligomers generated under conditions promoting fibril self-assembly. However, our arguments are general for *any* amyloid system in which oligomer formation is greatly accelerated by fibril surfaces (secondary association). In fact, even without this verification, it is likely that oligomer dissociation is fibril-mediated in most systems featuring secondary nucleation of fibrils. This follows since every system hitherto studied forms nonfibrillar oligomeric intermediates, so it is probable that secondary nucleation *always* works by accelerating their formation. Moreover, it is frequently speculated that primary nucleation of fibrils largely occurs at interfaces such as the air–water interface, lipid membranes, or at the sample container walls. Indeed this has been verified in several key amyloid-forming systems, including Aβ40,^27^ α-synuclein,^40,41^ hydrophobins,^42^ and IAPP.^43^ In such systems (and likely most amyloid-forming systems), our argument implies that “free” dissociation is *also* catalyzed by interfaces and largely occurs on the same interface responsible for primary nucleation.

We have shown that in situations where most oligomer formation is mediated by fibril surfaces, most dissociation must be, as well. Our conclusions are an inevitable consequence of the laws of thermodynamics in the absence of energy input, such as ATP-driven oligomer degradation. The alternative would imply a perpetual motion machine involving an endless reaction cycle from monomers to oligomers and back again via separate reaction pathways without any energy input to sustain it. To make our claims accessible to a wide audience, we have nonetheless provided significant amounts of experimental data to support them.

It is likely that Aβ42 can form fibrils with a variety of different morphologies depending on the reaction conditions, as has been observed for other proteins such as tau.^44^ If these different morphs have differing abilities to catalyze nucleation of new fibrils on their surfaces, as seems likely, then their ability to catalyze oligomer dissociation should also differ. The relationship between fibril morphology and oligomer formation and dissociation rates is an important topic for future research. In the case of Aβ42 and Aβ40, however, protocols for the reliable production of different fibril morphs of sufficient purity for kinetic analysis do not yet exist and will need to be developed before any such research can be carried out.

Our findings show that inhibitors that reduce the rate of oligomer production by binding to surfaces may also have valuable applications in an experimental context to study normally short-lived oligomeric species. For oligomers produced by either primary or secondary association, if, after a significant amount of oligomers have been formed, an inhibitor is added that blocks catalytic surfaces, the oligomers will be kinetically stabilized through the large reduction of their dissociation rate, enabling their characterization. Our experiments in Figure 2 demonstrate how effective this stabilization by removal of catalytic surfaces can be.

Finally, our results also highlight the importance of clearance, not only in the context of translating in vitro findings into living systems but also as a potentially crucial process to account for in drug development: increasing the clearance rate both raises the fibril mass concentration threshold for steady state and delays the attainment of any fixed fibril mass concentration. In other words, a higher clearance rate not only slows progression of oligomer buildup directly but also renders inhibitors of oligomer formation more potent at higher aggregate levels, suggesting a potential synergistic effect of combined therapies to target both these processes.

In summary, we have shown that Aβ oligomers formed during fibril self-assembly predominantly dissociate at the surfaces of Aβ fibrils and argued on theoretical grounds that this should be a general feature of amyloid oligomers generated during secondary association. This has fundamental implications for our understanding of amyloid oligomer chemistry and how to inhibit it. Failure to appreciate the crucial role of interfaces not just in oligomer formation but also in their dissociation may jeopardize future attempts to rationally design drugs to suppress the generation of toxic species in amyloid diseases.

## 4 Materials and Methods

### 4.1 Chemical Reaction Network for Aβ42 Self-Assembly

In ref (24), it was shown that the nucleation of new Aβ42 (and Aβ40) fibrils (concentration *P*; mass concentration *M*) from monomers (concentration *m*) requires first the formation of oligomeric intermediate species (concentration *S*) that are chemically distinct from fibrils. Subsequently to being formed, fibrils may elongate by monomer addition [rate 2*k*_+_*m*(*t*)*P*(*t*)]. Oligomers were shown to form both from free association of monomers [rate *k*_o1_*m*(*t*)^*n*^_o1_] and through a process mediated by the surfaces of existing fibrils [rate *k*_o2_*m*(*t*)^*n*^_o2_*M*(*t*)]. Subsequently, these oligomers convert at a slow rate to fibrils [rate *k*_conv_*m*(*t*)^*n*^_conv_]. Crucially, it was shown that oligomers also dissociate and that most oligomers ultimately dissociate rather than convert to fibrils.

In refs (23, 24, and 32,) it was left open whether dissociation occurs freely in solution [rate *k*_d1_*S*(*t*)] or whether it is fibril surface-mediated [rate *k*_d2_*M*(*t*)*S*(*t*)]. Explicitly considering both mechanisms, the full reaction network is then given by the following rate equations^23^
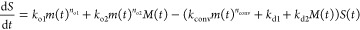
6
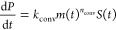
7
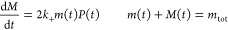
8(Rate constants are illustrated in Figure 1.)

The extent to which fibrils accelerate oligomer formation, *a*, can be quantified as the ratio of the rates for oligomer formation through secondary and primary association (Materials and Methods)
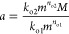
9

In “bulk” kinetic models, the formation of new fibrils from monomers occurs via coarse-grained primary and secondary nucleation reaction steps with rate laws *k*_*n*_*m*^*n*^_c_ and *k*_2_*m*^*n*^_2_*M*. The parameters from these rate laws are much more widely available than the rates of oligomer formation. Clearly, therefore, it would be advantageous to rewrite eq 9 in terms of these bulk kinetic parameters. Fortunately, this is possible using the relationships *n*_o1_ – *n*_o2_ = *n*_c_ – *n*_2_ and 3*k*_o2_/*k*_o1_ = 2*k*_2_/*k*_*n*_ derived in refs (23 and 24), yielding eq 1
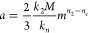


### 4.2 Analytical Solution for Inhibited Oligomer Kinetics with Constant Monomer Concentration

Including a clearance process in our model with rate constant *k*_cl_, the steady-state condition gives
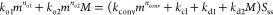
10

Under constant-monomer conditions, *M* grows indefinitely, so eventually this becomes
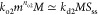
11
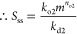
12i.e., the oligomer concentration plateaus. (Note that since fibril-binding inhibitors should reduce both fibril-mediated oligomer formation and fibril-mediated dissociation by an equal factor, such inhibitors have no effect on *S*_*ss*_.) To find the time scale on which this occurs, we now derive an approximate analytical solution to *S*(*t*) under constant-monomer conditions. The early time solution for *M* derived in ref (24) for conservation-of-mass conditions remains just as valid at early time here and is
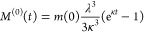
13
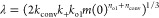
14
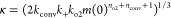
15

Now, under constant-monomer conditions and assuming that secondary processes are much faster than primary processes, the equation for oligomers becomes, for all but the earliest times
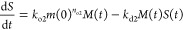
16
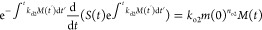
17

Integrating this gives us a self-consistent equation for *S*(*t*)
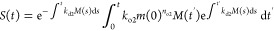
18
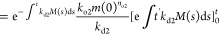
19
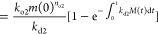
20

Using *M*^(0)^(*t*) as our starting point we have, for all but the earliest times *t* ≲ κ^–1^
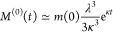
21
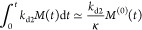
22
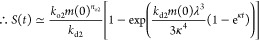
23

Examining this, we see that the oligomer concentration will initially grow exponentially before plateauing (as shown in Figure 4). The steady-state value is equal to the prefactor, so we may rewrite this as
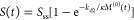
24

This allows us to calculate *S*_c_, the oligomer concentration attained at *M* = *M*_c_ = κ/*k*_d2_, as
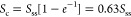
25

### 4.3 Kinetics of Fibril Mass Concentration *M* after the Plateau

Under constant-monomer conditions, once the oligomer concentration has plateaued at its steady-state value, new fibrils are produced at a constant rate
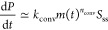
26

This is solved by *P* = *k*_conv_*m*(*t*)^*n*^_conv_*S*_ss_(*t* – *t*_0_) + *P*(*t*_0_), for some *t*_0_ > *t*_*p*_. The equation for *M* becomes
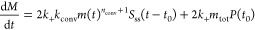
27

This is finally solved by
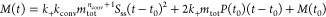
28

For times late enough after the plateau, this becomes quadratic, leading to significantly lower accumulation of fibrils compared to simply extrapolating the early time kinetics. This expression has no explicit dependence on *k*_o2_ or *k*_d2_. Therefore, since *S*_ss_ has previously been shown to be independent of inhibitor concentration, so is the fibril mass concentration *M*(*t*) after the plateau.

### 4.4 Expression and Purification of Peptides

The plasmid carrying synthetic genes with *E. coli*-optimized codons for Aβ42 wild-type PetSac, cloned by us^45^ as well as Aβ42-S8C (Pet3a, purchased from Genscript) were transformed into Ca^2+^ competent cells of *E. coli* strain BL21 DE3 pLysS star and the protein was expressed in autoinduction medium.^46^ The peptides were purified using ion-exchange chromatography as described with the minor change that lower salt concentration (50 mM NaCl) was used to elute the peptides, and size-exclusion chromatography (SEC) on a 26 × 600 mm Superdex 75 column was used instead of spin filters for base on molecular size. The ion exchange and SEC buffers for Aβ42-S8C contained 1 mM dithiothreitol (DTT) to avoid the dimerization of cysteines. The final SEC was performed in buffer without DTT in order to isolate the monomer and remove DTT from the sample prior to adding the label. The purified monomeric peptides were lyophilized as aliquots for further use.

### 4.5 Monomer Isolation Prior to Aggregation

The lyophilized aliquots of the purified WT Aβ42 were dissolved in 1 mL of 6 M GuHCl, 20 mM sodium phosphate, and 0.2 mM ethylenediaminetetraacetic acid (EDTA), pH 8.5 and subjected to gel filtration on a Superdex 75 10/300 column in 20 mM sodium phosphate buffer pH 8.0, with 0.2 mM EDTA. The middle part of the monomer peak was collected in a low-binding tube (Axygen) on ice and was typically found to have a concentration in the range of 20–80 μM (determined by absorbance of the collected part of the chromatogram peak using ϵ_280_ = 1400 L mol^–1^ cm^–1^). Alexa-488-labeled Aβ42-S8C aliquots were kept for thawing on ice in the dark to avoid photobleaching of Alexa.

### 4.6 Labeling of Purified Peptides with Alexa Fluor

Labeling of purified peptides with Alexa fluorophore was performed as described in ref (47). Briefly, lyophilized fractions were dissolved in 50 μL of Milli-Q water, yielding a peptide concentration of 14 μM. Alexa fluor 488 at a concentration of 3–4 mM in 20 μL of dimethyl sulfoxide was added to the dissolved peptide in order to have excess dye in the labeling mixture, which was kept overnight at 4 °C. The mixture was then added to 1 mL of 6 M GuHCl, 20 mM sodium phosphate, and 0.2 mM EDTA, pH 8.5 and subjected to SEC on a Superdex 75 10/300 column in 20 mM sodium phosphate buffer pH 8.0, with 0.2 mM EDTA. The absorbance at 280 as well as 488 nm was monitored using a Quadtech detector to follow the elution of the labeled peptide and excess dye as well as any unlabeled peptide, if present. The aliquots collected from the SEC were analyzed by sodium dodecyl sulfate-polyacrylamide gel electrophoresis and stored at −80 °C until further use.

### 4.7 Preparation of Oligomers for FCS

Freshly purified WT Aβ42 monomers were mixed with S8C-Alexa488 monomers in a 1:1.5 (labeled/unlabeled) ratio mix in 20 mM sodium phosphate, 0.2 mM EDTA buffer pH 8.0, with 6 μM thioflavin T. The total monomer concentration was close to 10 μM. The samples were pipetted into a 96-well plate (Corning 3881), 100 μL per well. The experiments were initiated by placing the 96-well plate at 37 °C. The reaction was stopped at half time of aggregation (*t*_1/2_) by removing the plate from 37 °C and immediately removing the fibrils by centrifuging for 2 min at 18,000 rpm. The fibrils are retained in the pellet, and the monomers and oligomeric species are in the supernatant. In order to separate the oligomers and monomers, the supernatant was injected into a gel filtration Superdex 75 10/300 column operated in 20 mM sodium phosphate buffer, pH 8.0, with 0.2 mM EDTA. The oligomeric fraction was collected and placed on ice, and the monomer peak was allowed to elute into the waste.

WT fibrils were prepared by adding freshly purified WT Aβ42 monomers (20 μM) in 20 mM sodium phosphate and 0.2 mM EDTA buffer pH 8.0, with 6 μM thioflavin T into a 96-well plate (Corning 3881), 100 μL per well. The experiments were initiated by placing the 96-well plate at 37 °C. Fibrils were removed when ThT fluorescence reached a plateau.

### 4.8 Fluorescence Correlation Spectroscopy

Oligomers alone (three replicates) or in the presence of WT fibrils (six replicates) were added to glass bottom dishes (Mattek Corporation, model P35G-1.5-10-C. Glass thickness #1.5). The total volume was 30 μL. Measurements were taken from distinct replicates.

FCS measurements were performed by placing the glass bottom dish at 37 °C on a Zeiss 980 confocal laser scanning microscope equipped for FCS, with a Zeiss water immersion objective, C-Apochromat 40*X*/1.2 NA. Samples were excited at 488 nm, and fluorescence emission was collected at 499–622 nm. Alexa 488 has a diffusion coefficient *D* = 414 μm^2^/s at 25 °C (Petrasek and Schwille, Biophysical Journal, 2008) which based on the Stokes–Einstein equation corresponds to *D* = 560 μm^2^/s at 37 °C due to the changes in temperature and viscosity. Together with a measured diffusion time τ_D_ = 26.1 μs at 37 °C gives a radius ω = 0.242 μm and a volume *V* = 0.59 fL for the FCS detection volume. Raw FCS curves are plotted in the Supporting Information.

The FCS autocorrelation curves were fitted to a three-component model (eq 29), where the components correspond to free dye, monomeric Aβ42-488, and oligomeric Aβ42-488, respectively.
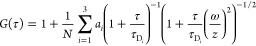
29

Here, *N* is the average number of particles in the detection volume, *a*_*i*_ is the fractional amplitude of each component, τ_D_*i*__ is the mean diffusion time through the focus, and ω and *z* are the distances from the detection focus center in the radial and axial directions, respectively, where the detected intensity has decreased by a factor *e*^–2^. Note that we fix the diffusion times when fitting to 26.1, 90, and 300 μs, corresponding to free dye, monomeric Aβ42-488, and oligomeric Aβ42-488.

The fractional amplitudes of the three components, *a*_*i*_, scale with the square of the fluorescence brightness of the corresponding component. Therefore, they do not correspond to the relative concentrations of the components in the sample but to the relative contribution of each component to the autocorrelation curve. By comparing mean diffusion rates of oligomers and monomers, we find that the average oligomer consists of approximately 37 monomers irrespective of the fibril concentration. We separately estimate that oligomers are on average 10 times brighter than monomers due to partial quenching and to selective enrichment of unlabeled monomers in oligomers. Combined, these numbers enable us to convert the fractional amplitudes into relative mass concentrations, as plotted in Figure 2. Note that even large errors in these numbers will not alter the fundamental conclusion that oligomers dissociate much more rapidly in the presence of fibrils because they affect the concentrations of oligomers with and without fibrils by the same factor. The results of the fitting and the quenching estimation are provided in a Supporting Information spreadsheet. Standard statistical tests indicated these results to have a high statistical significance (see below).

The earliest measurements that could be taken were 3–7 min after initiation of the experiments. In the absence of fibrils, most protein mass is still oligomeric at this time. In the presence of fibrils, however, the mass fraction of oligomers has already attained close to its ultimate value, i.e., the dissociation reaction is already approaching equilibrium. It was thus not necessary to take additional time points, and it can be inferred that the oligomer dissociation half-life is less than 7 min for these samples.

### 4.9 Preparation of Oligomers for μFFE

Labeled and nonlabeled monomeric protein was mixed at a 1:3.5 ratio for a total final concentration of 10 μM (note: this ratio was different from that used in the FCS experiments to help demonstrate the robustness of our conclusions to different reactants). The mixture was loaded in a 96-well half area low-binding PEG-coated polystyrene plate with a transparent bottom (3881 Corning). The sealed plate was incubated at 37 °C without shaking in an FLUOstar Omega plate reader (BMG Labtech, Offenburg, Germany). The aggregation of the sample was monitored by following the quenching of the Alexa 488 fluorophore upon fibril formation.

When the aggregation reached the beginning of the plateau, all samples were retrieved and pulled together. This time point is later than that used in the FCS experiments since the resulting much lower concentration of monomers improves resolution of oligomers in μFFE. It also helps ensure the generality of our findings regarding the mechanism of oligomer dissociation. To study the direct dissociation of the oligomers, 400 μL aliquots were taken and subjected to centrifugation at 15,000 rpm for 15 min to remove the majority of the fibrils. 200 μL of the supernatant was taken and diluted to half with 200 μL of sodium phosphate buffer. For the study of the fibril-mediated dissociation, 200 μL aliquots were taken and diluted to half with sodium phosphate buffer. Thus, unlike in FCS, the fibrils present are labeled. After 3 h of incubation at room temperature, all samples were subjected to 15 min of centrifugation at 15,000 rpm to ensure removal of fibrils, which could interfere with the measurement. 200 μL from the supernatant was then diluted to an approximate final aβ 42 concentration of 1 μM and a sodium phosphate concentration of 10 mM.

### 4.10 Microfluidic Device Fabrication

Devices were designed by using AutoCAD software (Autodesk) and photolithographic masks printed on acetate transparencies (Micro Lithography Services). Polydimethylsiloxane (PDMS) devices were produced on SU-8 molds fabricated via photolithographic processes as described elsewhere^48^ with UV exposure performed with custom-built light-emitting diode-based apparatus.^49^ Following development of the molds, feature heights were verified by a profilometer (Dektak, Bruker) and PDMS (Dow Corning, primer and base mixed in 1:10 ratio) was applied and degassed before baking at 65 °C for 1.5 h. Devices were cut from the molds, and holes for tubing connection (0.75 mm) and electrode insertion (1.5 mm) were created with biopsy punches, cleaned through sonication in IPA, and bonded to glass slides using oxygen plasma. Before use, devices were rendered hydrophilic via prolonged exposure to oxygen plasma.^50^

### 4.11 μFFE Device Operation

Liquid-electrode microchip free-flow electrophoresis (μFFE) devices were operated as described previously.^51^ Briefly, fluids were introduced to the device by PTFE tubing, 0.012 in. ID × 0.030 in. OD (Cole-Parmer) from glass syringes (Gas Tight, Hamilton) driven by syringe pumps (Cetoni neMESYS). μFFE experiments were conducted with auxiliary buffer, electrolyte, and sample flow rates of 1000, 200, and 10 μL h^–1^, respectively. Potentials were applied by a programmable benchtop power supply (Elektro-Automatik EA-PS 9500-06) via bent syringe tips inserted into the electrolyte outlets. All experiments were performed on a custom-built single-molecule confocal fluorescence spectroscopy setup equipped with a 488 nm wavelength laser beam (Cobolt 06-MLD 488 nm, 200 mW diode laser, Cobolt). Photons were detected by using a time-correlated single-photon counting (TCSPC) module (TimeHarp 260 PICO, PicoQuant) with a time resolution of 25 ps.

Using a custom-written script, single-molecule events were recorded as discrete events using a Lee filter of 3 from the acquired photon stream as fluorescence bursts with 0.001 μs of the interphoton time and containing 22 photons minimum. Using these parameters, the single-molecule bursts and their intensities were reported as functions of device position. Oligomer bursts were distinctly characterized by a higher photon intensity detected per molecule than monomeric protein.^52^ The analysis and resulting oligomer and monomer counts are provided in a Supporting Information spreadsheet.

### 4.12 Statistical Hypothesis Testing

It is obvious from the data presented in Figure 2 that the concentration of oligomers relative to that of monomers is far lower after incubation with fibrils. To confirm this beyond doubt, a one-tailed unequal variance *t*-test was performed. This yielded a *p* value of 0.00028 for the FCS data (*t* statistic 6.83 and degrees of freedom 5.77); therefore, the null hypothesis (mean oligomer mass fraction in the presence of fibrils ≥mean in the absence of fibrils) is extremely unlikely and can be rejected to a confidence level >99.9%. For the μFFE data, it yielded a *p* value of 0.00083 (*t* statistic 7.55 and degrees of freedom 2.39); therefore, the null hypothesis (mean oligomer number fraction in the presence of fibrils ≥mean in the absence of fibrils) is very unlikely and can be rejected to a confidence level >99.5%.

## Supporting Information Available

Raw FCS curves and mathematical calculations of time scales (PDF)
FCS data analysis (XLSX)
μFFE data analysis (XLSX)
