# Supplementary material for: Aβ Oligomer Dissociation Is Catalyzed by Fibril Surfaces
Source: ACS Chem Neurosci. 2024 May 24;15(11):2296–307. doi: 10.1021/acschemneuro.4c00127 (PMC11157482; doi:10.1021/acschemneuro.4c00127)
Supplement: Supplementary file 1 — cn4c00127_si_001.pdf [file cn4c00127_si_001.pdf]

Supporting Information: Aβ oligomer dissociation is catalyzed by fibril surfaces

Alexander J. Dear,[†,‡] Dev Thacker,[†] Stefan Wennmalm,[¶] Lei Ortigosa-Pascual,[†] Ewa A. Andrzejewska,[‡] Georg Meisl,[‡] Sara Linse,[†] and Tuomas P. J. Knowles[*,‡,§]

†*Department of Biochemistry and Structural Biology, Lund University, Sweden*

‡*Centre for Misfolding Diseases, Department of Chemistry, University of Cambridge, Lensfield Road, Cambridge CB2 1EW, United Kingdom*

¶*Department of Applied Physics, Biophysics Group, SciLifeLab, Royal Institute of Technology-KTH, 171 65 Solna, Sweden*

§*Cavendish Laboratory, University of Cambridge, J J Thomson Avenue, Cambridge CB3 0HE, UK*

E-mail: tpjk2@cam.ac.uk

S1 Raw FCS curves

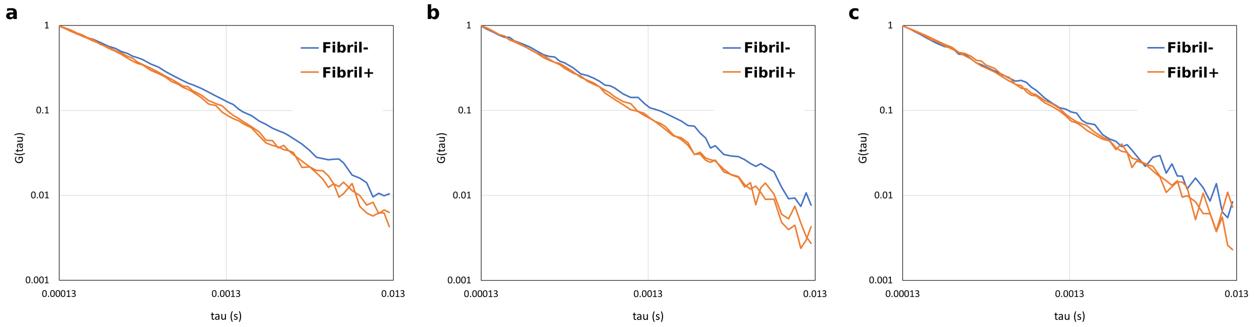

Figure S1: Raw FCS curves, plotted in groups of 3 for clarity. The curves in panel **c** featured the lowest fibril mass concentrations (50 and 200 nM, compared to 400 and 1000 nM in panels **a** and **b**). In all panels a clear difference is visible between the curve without fibrils and the curves with fibrils. The results of fitting these curves to establish the proportion of monomers and oligomers, available as a supplementary spreadsheet, were found to have high statistical significance (see Methods.) The smaller difference between curves with and without fibrils in panel **c** was revealed to be more a consequence of higher free dye levels in the corresponding samples than of the lower fibril mass concentrations.

S2 Oligomer vs fibril plateau timescales

The plateau time t_p, defined as the time at which the oligomer concentration has reached half of its final value, is given as:

$$\frac{1}{2} = \exp\left(\frac{k_{\mathrm{d2}} m(0) \lambda^3}{3\kappa^4}\left(1 - e^{\kappa t_p}\right)\right) \tag{S1}$$

$$\ln(2) \simeq \frac{k_{\mathrm{d2}} m(0) \lambda^3}{3\kappa^4} e^{\kappa t_p} \tag{S2}$$

$$t_p = \frac{1}{\kappa} \ln\left[\frac{3\ln(2)\kappa^4}{k_{\mathrm{d2}} m(0) \lambda^3}\right]. \tag{S3}$$

We may compare this to the half-time for fibril formation under constant-monomer conditions:

$$2 \simeq \left(1 + \frac{e^{\kappa t_h}}{c}\frac{\lambda^3}{3\kappa^3}\right)^c \tag{S4}$$

$$2^{1/c} - 1 \simeq \frac{e^{\kappa t_h}}{c}\frac{\lambda^3}{3\kappa^3} \tag{S5}$$

$$t_h = \frac{1}{\kappa}\ln\left[c(2^{1/c} - 1)\frac{3\kappa^3}{\lambda^3}\right]. \tag{S6}$$

At $m(0) = 5$ μM we obtain $\kappa \simeq 8$ h^{-1}. k_{d2} can be calculated from the main text result for Aβ42 $k_{\mathrm{d2}}M/k_{\mathrm{d1}} = 30 \cdot M$ and reported fitting results from ref. (S1) as $\simeq 0.07$ h^{-1}. Since $c \simeq 0.6$, we then see that the oligomer concentration under steady-state-monomer conditions plateaus long after the closed fibril assembly reaction completes.

References

(S1) Michaels, T. C. T., Šarić, A., Curk, S., Bernfur, K., Arosio, P., Meisl, G., Dear, A. J., Cohen, S. I. A., Dobson, C. M., Vendruscolo, M., Linse, S., and Knowles, T. P. J. (2020) Dynamics of oligomer populations formed during the aggregation of Alzheimer's Aβ42 peptide. *Nat. Chem. 12*, 445–451.
